# Very late recurrence of sinus of Valsalva aneurysm rupture after patch repair

**DOI:** 10.1186/1471-2482-14-73

**Published:** 2014-10-01

**Authors:** Ting-Tse Lin, Hsiao-En Tsai, Lin Lin, Tsung-Yan Chen, Cheng-Pin Lee, Chih-Chen Wu

**Affiliations:** 1 Division of Cardiology, Department of Internal Medicine, National Taiwan University College of Medicine and Hospital Hsin-Chu Branch, No.25, Ln. 442, Sec. 1, Jingguo Rd., North Dist., Hsinchu City 300, Taiwan; 2 Department of Surgery, National Taiwan University College of Medicine and Hospital Hsin-Chu Branch, Hsin-Chu City, Taiwan

**Keywords:** Ruptured Sinus of Valsalva aneurysm, Patch repair, Left-to-right shunt

## Abstract

**Background:**

Sinus of Valsalva aneurysm (SVA) is an uncommon cardiac defect accounting for only 1% of congenital cardiac anomalies and the most common complication is ruptured into the atrium or ventricle. Very late recurrence of ruptured SVA after patch repair is extremely rare.

**Case presentation:**

We present a case of 57-year-old man had received repair for ruptured Sinus of Valsalva aneurysm at 19 ages. In the clinics, he presented with exertional dyspnea and leg swelling. The serial examination disclosed he had bicuspid aortic valve and very late rupture of SVA connecting to right atrium. After surgical repair again, he was discharged smoothly.

**Conclusion:**

A very late recurrence of ruptured SVA after surgical repair was rare. We reported a case with unique echocardiographic presentation and a successful repair.

## Background

Congenital sinus of Valsalva aneurysm (SVA) is caused by a deficiency of normal elastic tissue and abnormal development of the bulbus cordis [1]. Approximately 65-85% of SVA originate from the right sinus of Valsalva, while SVA originating from noncoronary (10-30%) and left sinuses (<5%) are exceedingly rare [2]. The epidemiology showed SVA is more prevalent in Asian surgical series (0.46-3.5%) [3]. This report presents the sequel of repeated rupture of previously repaired SVA.

## Case presentation

**Figure 1:**
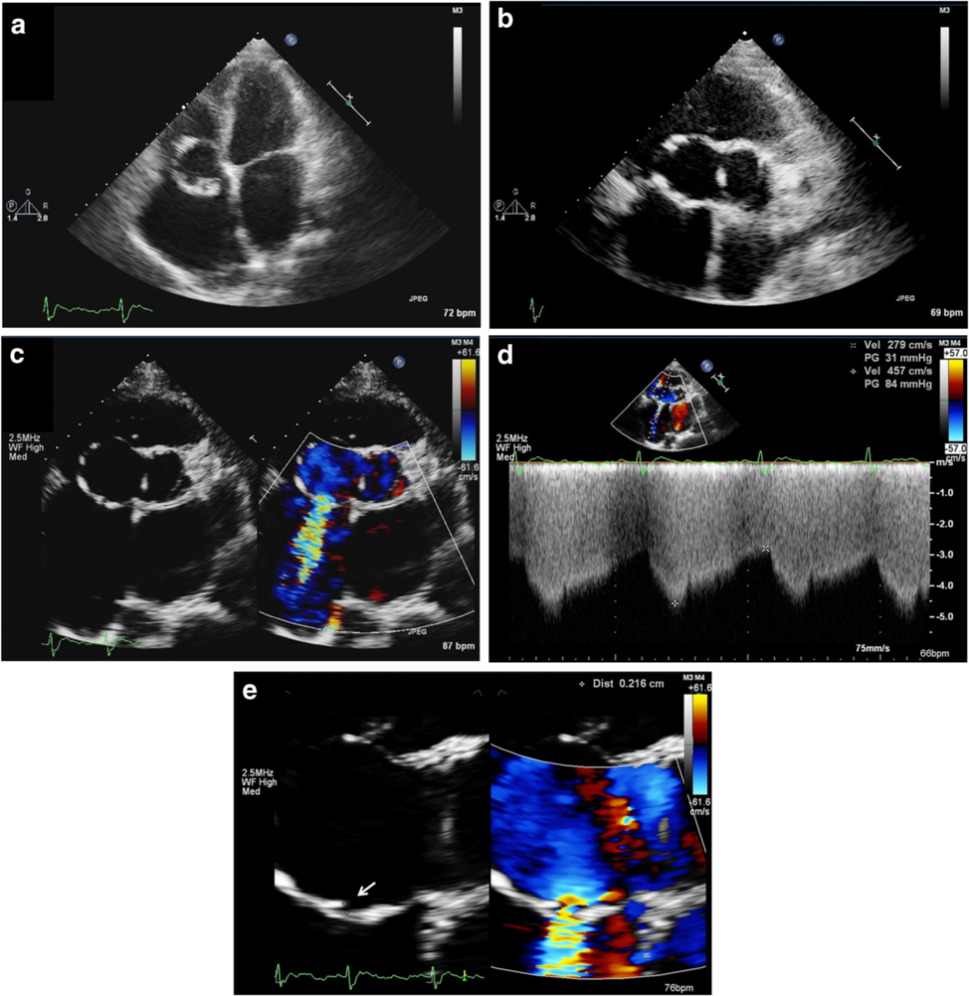
**Transthoracic echocardiography of ruptured sinus of Valsalva aneurysm. a**. Apical four chamber view of transthoracic echocardiography reveals an aneurysm with thick and hyperechoic wall at the level of tricuspid ring. **b**. Parasternal short-axis view shows bicuspid aortic valve and an aneurysm (SVA) protruding from right coronary cusp to the right atrium. **c**. In the same view, color Doppler image shows shunting from SVA into right atrium. **d**. Continuous wave Doppler demonstrated the continuous flow. **e**. Zoom-in view from figure 1c revealed the defect (*arrow*) at the posterior aspect of the SVA. SVA indicates sinus Valsalva aneurysm; RA, right atrium; Av, aortic valve; LA, left atrium.

**Figure 2:**
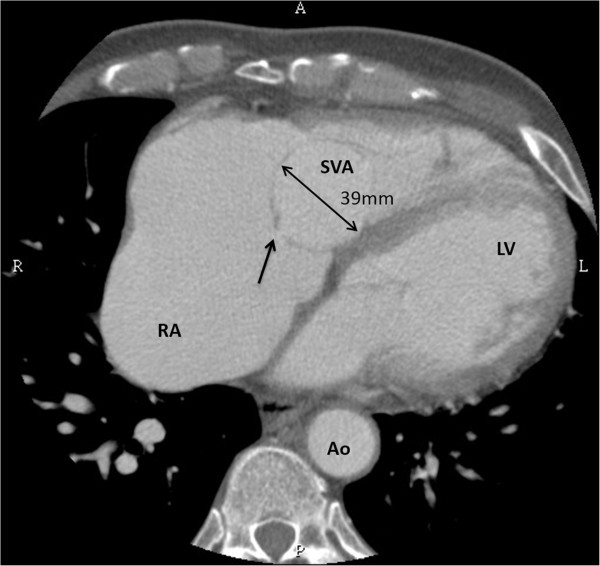
**Computed tomography showed a defect (*arrow*) from aneurysm (An) to right atrium (RA).** LV indicates left ventricle; Ao, aorta.

**Figure 3:**
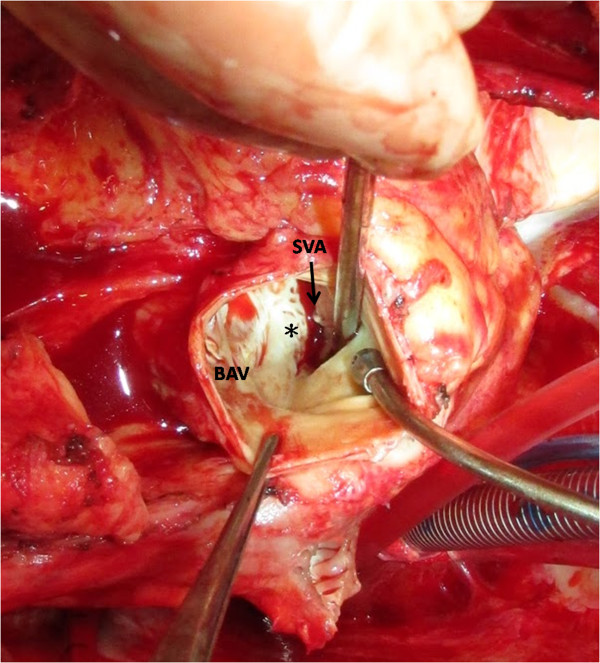
**View from transected aorta. It shows bicuspid aortic valves with a large cavity protruding from right sinus of Valsalva.** BAV indicates bicuspid aortic valve; SVA, sinus Valsalva aneurysm; asterisk (*), previous suture.

**Figure 4:**
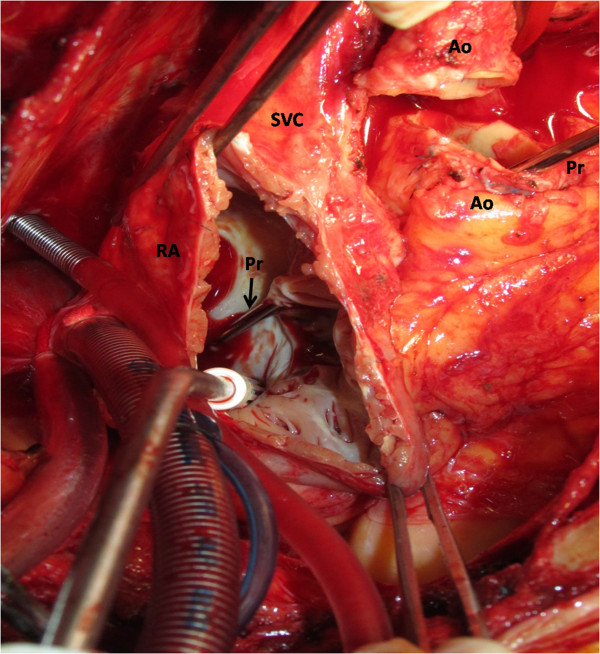
**View from incised right atrium.** The probe from transected aorta can go through the fistula to right atrium. SVC indicates superior vena cava; Ao, aorta; RA, right atrium; Pr, probe.

A 57-year-old man presented with dyspnea on exertion and leg swelling for four months. He had undergone surgical repair of ruptured right SVA by direct closure at age of 19, and remained asymptomatic after the operation. On physical examination, a grade 3/6 continuous murmur was heard at his right lower sternal border, with increasing intensity while he was leaning forward. Electrocardiogram (ECG) showed atrial flutter with 2:1 atrioventricular conduction (ventricular rate around 100 beats per minute) and complete right bundle branch block. Chest X ray revealed enlargement of right atrium and ventricle, but no pulmonary congestion. Transthoracic echocardiography revealed bicuspid aortic valve and a large cystic structure protruding from right sinus of Valsalva to the right atrium. The wall of the cystic structure was relatively thick and hyperechoic, which was distinct from the congenital form of SVA (Figure 1a, b). Color duplex showed a continuous shunt from the aorta to the right atrium through a small defect at the posterior aspect of the aneurysm (Figure 1c, d, e and Additional file 1: Movie 1). Gradual bulging of the previous patch sutured at the SVA 38 years ago with recurrent rupture was impressed. Multidetector computed tomography also demonstrated a large SVA with a defect connecting to a dilated right atrium (Figure 2).Surgical exploration from both right atrial side and aortic side confirmed the communication between SVA and the right atrium (Figures 3 and 4). The defect was located at a weak point of suture between the previous patch and the sinus of Valsalva. Double patch method was performed with bovine pericardium patches. From the aortic side a patch was sutured from the aortic annulus to the inferior rim of the right coronary artery; and from the right atrial side the redundant sac of the SVA was resected and the defect was repaired with another patch. The patient was discharged uneventfully two weeks after surgery.

## Conclusion

SVA is an uncommon cardiac defect, with higher incidence in Oriental than in Caucasian patients [3]. Patients who underwent surgical repair of ruptured SVA had 95% overall survival at 20 years [4]. In one series reviewing 31 patients undergoing surgical correction of ruptured SVA, only two of them needed reoperation for closure of recurrent fistula during a mean follow-up period of 25 years [4]. In another series of 81 patients of SVA (34% ruptured), there were no reports of recurrent rupture during follow-up (3 months to 40 years, mean 4 years) [5]. We report this very late recurrence of ruptured SVA 38 years after the first surgical repair, presented with heart failure symptoms, with its unique echocardiographic pictures.

## Consent

Written informed consent was obtained from the patient for publication of this Case report and any accompanying images. A copy of the written consent is available for review by the Editor of this journal.

## Abbreviations

ECG: Electrocardiography; SVA: Sinus of Valsalva aneurysm.

## Competing interests

The authors declare that they have no competing interests.

## Authors' contributions

TT Lin made substantial contribution to conception and drafted the manuscript. HET carried out the surgical repai. LL participated in the diagnosis and performed analysis and interpretation of the images. TYC and CPL made the contribution for acquisition of data. CCW revising it critically for important intellectual content. All authors read and approved the final manuscript.

## Pre-publication history

The pre-publication history for this paper can be accessed here:

http://www.biomedcentral.com/1471-2482/14/73/prepub

## Supplementary Material

Additional file 1 **Movie 1.** Parasternal short-axis view shows bicuspid aortic valve and an aneurysm protruding from right coronary cusp to the right atrium, with continuous shunting from aneurysm into right atrium through a small defect. Click here for file
